# The Growth and Scope of Voluntary Hospitals in London

**Published:** 1897-07-31

**Authors:** 


### The Institutional Workshop.

### THE GROWTH AND SCOPE OF VOLUNTARY HOSPITALS IN LONDON.

(From a Correspondent.)

### II.—THE SPREAD OF GENERAL HOSPITALS IN THE EIGHTEENTH CENTURY.

At the root of all the new charitable efforts of the eighteenth century lay that great principle of co-operation which then first began to stir in the minds of Londoners and make its earliest essays in the formation of banks and trading companies. Sir Thomas Bernard, one of the ablest philanthropists of early days, has well defined this principle: "When the exertions of many individuals are directed by one spirit to one object, they acquire a momentum of power which never can be attained by an unconnected individual, not even by the administration of government itself. They have one single point in view to which they devote all their efforts; they act with a degree of zeal and perseverance, transmitted in corporate succession, and ever attending the emulation of many labouring disinterestedly and strenuously in a common cause. To a great and extended kingdom it is of infinite benefit that its members should be habituated to co-operate for these purposes, and to devote a part of their time and attention to the well-being of their fellow-subjects. Uniting the opinion and concentrating the confidence of many, a single individual may be equal to anything. A centre of action being thus obtained, man may acquire the same interest in the happiness of others that he possesses in his own; and the best and purest species of public spirit may be generated and prepared in a great country."

No finer definition could be given of the inspiring force to which voluntary hospitals owe their successful development. When the results of official effort in this and other countries are considered, it may be confidently claimed that no such success, no such continued progress, would have been witnessed in the history of hospital work had Government accepted, from the first, full responsibility for the care of the sick poor, and exacted by taxation those sums for their maintenance which now for nearly two hundred years have been freely offered by the public for works of mercy privately organised. During this period voluntary hospital work has proved, as no State institution could ever be, a powerful link between rich and poor. It stood firm and spread its healing branches over the toiling and oppressed population of London, at times when legislation was brutally stern in repression but powerless to redress abuses. It brought hope to countless sufferers and peace to many a dying bed, when the Church was passing through its deadest season of inactivity; and during periods of public excitement and class bitterness the voluntary hospitals have been the highest, almost the only, witness of the true spirit of brotherhood still subsisting among Londoners.

The work of ministering by private effort to the need of London was even then a mighty one, and has, perhaps, never yet been adequately performed. After the establishment of Westminster Hospital three populous districts were fairly well provided for, St. Bartholomew's, in Smithfield, ministered to the great central population of the busiest part of the town, and St. Thomas's, close on the Surrey side of London Bridge, stood readily accessible to the crowded quarters on either side of the river.

The year after the founding of Westminster Hospital, Thomas Guy planned the institution, completed in 1724, which bears his name, modelling it to a great extent on the constitution of the Royal hospitals. The government was vested in the hands of 60 governors, from whom a committee of 21 was chosen for the actual despatch of business. Sufficient money was bequeathed to defray all expenses of building, and leave an ample annual income, so that no subscribers were needed, and to all appearance the affairs of the hospital were secured from dependence on the generosity of succeeding generations. Guy's Hospital can therefore hardly be held to form part of the voluntary system. His old attachment to St. Thomas's, on which he had bestowed many rich benefactions, led him to fall into the error, which has since been more than once repeated, of establishing his institution in the immediate neighbourhood of the older hospital. The fact that the wards of St. Thomas's were overcrowded, and that many applicants found their way there from a distance, no doubt induced a belief that the actual situation, if airy and good, was of no importance to the poor, who could make their way thither without great difficulty from all quarters of the town. It took many years of experience and close observation to show that by far the greater proportion of those who benefit from a hospital are persons residing within a radius of a mile or two from its gates, and that the usefulness of both institutions is seriously impaired when a new undertaking is planted down in the immediate vicinity of another having similar aims. It is hardly possible that the inconvenience was not felt from the beginning, while later on, owing to the growth of the medical schools, the evil became more serious. Mr. Walker, late steward of St. Thomas's Hospital, in giving evidence before the Lords' Committee in 1891, stated: "In the olden times at St. Thomas's, when the two hospitals—Guy's and St. Thomas's—were together and could make up a thousand beds between them, we had to compete for accidents or to bribe the police to bring them to us." It is only necessary to point out that the error was that of an individual, moved by local attachments.

In less than ten years after the building of Guy's, hospital development made another advance. It became necessary to find better quarters for the newly-established hospital in Westminster, the original premises being peculiarly ill adapted for the purpose. Two sites were suggested, and the majority decided on a house in Castle Lane in the lower part of Westminster, to which the hospital was accordingly removed. But the other site offered such overwhelming advantages in the eyes of the physicians and surgeons and many of the early governors that they seceded one and all from the original undertaking, and founded St. George's Hospital in the house of Lord Lanesborough at Hyde Park Corner. It was an unrivalled position. South and west stretched fields and lanes leading to the villages of Pimlico, Chelsea, and Kensington.

Eastward lay the slopes of the Green Park, and far in front stretched the bare plain, principally used for exercising cavalry, of Hyde Park. The project was liberally supported, and the promoters commanded so much public confidence that patients were received within three months of the scheme being set on foot, in January, 1834. As the hospital stood practically in the country, all the patients were supplied, comparatively speaking, from a distance, and it early acquired the character it still to a less extent retains, of serving as a refuge in time of illness to the domestic servants of the metropolis. This class of persons was, indeed, in peculiar need of aid. The wages of indoor servants were low; many of them came to service from distant homes in the country, and on the approach of illness, being subject to instant dismissal, they were exposed to even greater destitution, from want of friends or knowledge of the town, than the working population of London. St. George's Hospital was fortunate in attracting from the beginning an unusually strong revenue from annual subscribers; a cordial feeling was cultivated with the earlier hospital in Westminster, and a healthy spirit of emulation took the place of any bitterness or unfair rivalry.

The London Hospital, founded in 1740 in a district rapidly growing into importance with the growth of manufactories and the shipping trade, owes its origin to the zeal of its first surgeon, John Harrison. The site on which the building was finally fixed on the road between Whitechapel and Bow was at that time surrounded by open ground, and well fitted for the purpose. It was within convenient reach of the densely-populated district of East Smithfield, then inhabited by "persons of the very lowest description;" a mile to the south of the hospital along the banks of the river lay crowded a shifting set, fast increasing as trade progressed, of seamen and casual labourers, among whom dangerous accidents were daily and hourly occurring, while in the neighbourhood of Spital Field and Bethnal Green, just a little to the north, were large manufactories, attracting a fixed working population, and contributing with fatalistic indifference to all precautions in favour of safety and health, more than their necessary share of casualty and disease. From the first moment there was never any doubt about the necessity for the hospital or its success. The profession of the founder and the character of its surroundings determined its surgical bias, and although medical work and sufferers from disease were not neglected, the surgical part of the undertaking was for many years the most conspicuous. The hospital was not regularly incorporated till some years later, when its constitution was based on the usual lines of voluntary support and control. It met with hearty support from the wealthy, and large sums were speedily forthcoming for its maintenance and partial endowment.

One more general hospital owes its foundation to this period. In 1745 the Middlesex Hospital was instituted for the reception of "sick and lame patients," and two years later its plan was enlarged to make provision for the relief of lying-in married women. The same course was followed as in the case of St. George's, and the new building was located on the outskirts of London, in a region which hardly at the commencement of the present century, fifty years later, had began to suffer under what a quaint writer of the time described as the "cacoethes ædificando" of modern days. Oxford Street had then only a few houses on the north side, and is described as "a deep hollow road and full of sloughs, with here and there a ragged house, the lurking-place of cut-throats." The Berners Street of to-day was in a district of spreading fields and waste ground, with the village of "Marybonne" not far distant, and a clear stretch beyond up to the low range of Highgate Hills. The new hospital may have been resorted to by some of the least wild from the crowded district of St. Giles's, where the inhabitants are stated to have been "immersed in the very extreme of wretchedness, dissolute and drunken, a nest of banditti"; but more probably the patients were drawn from the civilised working population in the district east of Regent Street. As with all other London hospitals, however, no limit was set to the district from which patients were received.

There were no further additions to the general hospitals of London for over seventy-five years. The plan of arrangement with reference to locality, as it then existed, was probably the best that could have been devised for the population. Westminster on the south, St. George's on the west, Middlesex on the north (but too far west for symmetry), and the London Hospital on the east embraced the entire metropolis. St. Bartholomew's was situated in the centre of the city, and on the Surrey side of the river were Guy's and St. Thomas's. Our predecessors in hospital distribution set about their work it will be seen, in no haphazard fashion.

Their further efforts to bring medical aid to their poor neighbours were continued on an even more systematic plan.

